# Tape nanolithography: a rapid and simple method for fabricating flexible, wearable nanophotonic devices

**DOI:** 10.1038/s41378-018-0031-4

**Published:** 2018-10-08

**Authors:** Qiugu Wang, Weikun Han, Yifei Wang, Meng Lu, Liang Dong

**Affiliations:** 1 Department of Electrical and Computer Engineering, Iowa State University, Ames, IA 50011 USA; 2 Department of Mechanical Engineering, Iowa State University, Ames, IA 50011 USA; 3 Microelectronics Research Center, Iowa State University, Ames, IA 50011 USA

## Abstract

This paper describes a tape nanolithography method for the rapid and economical manufacturing of flexible, wearable nanophotonic devices. This method involves the soft lithography of a donor substrate with air-void nanopatterns, subsequent deposition of materials onto the substrate surface, followed by direct taping and peeling of the deposited materials by an adhesive tape. Without using any sophisticated techniques, the nanopatterns, which are preformed on the surface of the donor substrate, automatically emerge in the deposited materials. The nanopatterns can then be transferred to the tape surface. By leveraging the works of adhesion at the interfaces of the donor substrate-deposited material-tape assembly, this method not only demonstrates sub-hundred-nanometer resolution in the transferred nanopatterns on an area of multiple square inches but also exhibits high versatility and flexibility for configuring the shapes, dimensions, and material compositions of tape-supported nanopatterns to tune their optical properties. After the tape transfer, the materials that remain at the bottom of the air-void nanopatterns on the donor substrate exhibit shapes complementary to the transferred nanopatterns on the tape surface but maintain the same composition, thus also acting as functional nanophotonic structures. Using tape nanolithography, we demonstrate several tape-supported plasmonic, dielectric, and metallo-dielectric nanostructures, as well as several devices such as refractive index sensors, conformable plasmonic surfaces, and Fabry-Perot cavity resonators. Further, we demonstrate tape nanolithography-assisted manufacturing of a standalone plasmonic nanohole film and its transfer to unconventional substrates such as a cleaved facet and the curved side of an optical fiber.

## Photonics: Nanolithography for flexible devices

Tape nanolithography enables the rapid and low-cost fabrication of a range of photonic devices. The use of tape to facilitate the transfer of patterns from a substrate is known to provide a versatile and simplistic approach for fabricating uniform patterns. Now, a team led by Liang Dong at Iowa State University, USA, deposits metal and dielectric films onto a nanoscale, prepatterned PDMS substrate, and then applies a tape to remove the patterned surface for subsequent transfer. Using this approach various nanopatterned surfaces are demonstrated, including their application in devices such as refractive index sensor, plasmonic surface and Fabry-Perot cavity resonator, all of which are flexible and thus could be used for conformable electronics and photonics.

## Introduction

Nanotransfer printing has been demonstrated to be a cost-effective, high-throughput method for producing various nanopatterns^1–6^. This technique has enabled the transfer of nanopatterned films from a stamp to a variety of flexible or stretchable substrates to realize inexpensive thin-film transistors^7,8^, integrated circuits^9,10^, epidermal electronics^11,12^, surface-enhanced Raman spectroscopy substrates^13,14^, negative-index metamaterials^15,16^, and microelectromechanical devices^17–19^. Notably, adhesive tapes have been used to exfoliate two-dimensional (2D) materials such as graphene^20^ and molybdenum disulfide^21^ and have been incorporated in the nanotransfer printing process as an intermediate transfer medium or a sacrificial layer. Additionally, because the adhesion strength of some adhesives dramatically decreases at elevated temperatures, thermal release tapes have enabled the transfer of different materials and devices to target substrates (e.g., nanotube transistors from quartz to plastic substrates^22^). With the help of adhesive polymers whose adhesion strength can be controlled by a plasticizing solvent, the on-demand release of nanowires from tape has been used to transfer nanowire-based transistors to supporting materials^23^. Apart from functioning as sacrificial layers, adhesive tapes have also been used to planarize nanopatterned substrates to generate large-area nanogaps^24,25^ and to serve as substrates for the transfer of aluminum nanoholes from compact discs under critical temperatures^26,27^. Therefore, existing tape transfer methods have significantly facilitated the transfer processes of various nanopatterns where the tapes function as temporary sacrificial layers or device substrates. The abilities and versatilities of the tape transfer methods, however, are not currently fully exploited for flexible and wearable optic solutions for emerging applications. Additionally, although relatively complex and sophisticated procedures are required to preform the nanopatterns on a donor substrate for tape transfer, the transferred nanopatterns are often limited to a simple shape or a single layer, thus limiting the possibilities for more applications. In addition, there have been very few explorations regarding the new properties of and applications for the remaining structures and materials on donor substrates after the transfer of nanopatterns.

This paper reports a novel tape nanolithography method for realizing plasmonic, dielectric, and metallo-dielectric nanostructures on the surfaces of prepatterned donor substrates, transferring these nanostructures to the tape surface, and simultaneously realizing inverse nanostructures embedded in the donor substrate with shapes complementary to those transferred to the tape surface. This method enables the rapid and economical production of various flexible and wearable nanophotonic structures and devices. We demonstrate that the tape nanolithography method allows the rapid manufacturing of many tape-supported nanophotonic devices containing nanopatterned metallic or dielectric materials, metal-dielectric-metal (MIM) stacks, or all-dielectric multilayered films. We also demonstrate the production of flexible refractive index sensors, conformable plasmonic surfaces, and Fabry-Perot (F–P) cavity resonators using tape nanolithography. Further, by dissolving or detaching the supporting tape, standalone nanopatterned films are realized and transferred to irregularly shaped surfaces, such as the tips and side surfaces of optical fibers.

## Results

**Fig. 1:**
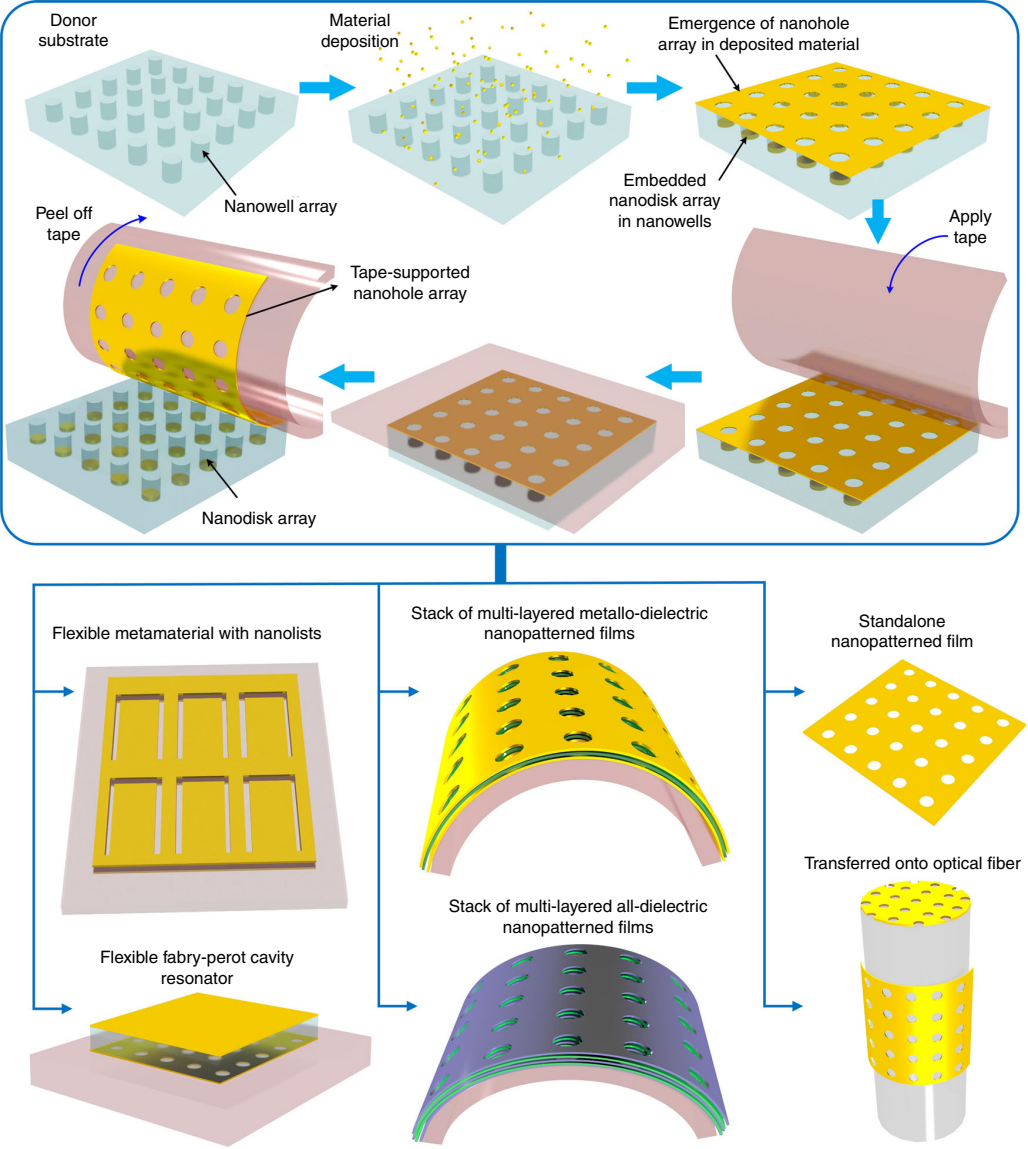
Schematic of tape nanolithography for producing nanopatterned films and transferring them to the surfaces of adhesive tape and other substrates. An array of nanoholes emerges in a metallic thin film when the material is deposited on the surface of a polydimethylsiloxane (PDMS) donor substrate with preformed nanowells. The tape is then applied to the substrate to transfer the nanopatterned films to the tape surface. By using tape nanolithography, many nanophotonic structures and devices can be formed on the tape surface, such as multilayered metallo-dielectric and all-dielectric nanopatterned films, flexible metamaterials, and F–P cavity resonators, and nanohole films can be formed at different locations on optical fibers

As described in Fig. 1, tape nanolithography involves depositing a single layer or a multilayer stack of thin films onto the surface of a donor substrate (e.g., polydimethylsiloxane or PDMS) containing preformed air-void nanopatterns and then peeling the deposited materials from the donor substrate using an adhesive tape. The nanopatterns automatically emerge in the deposited materials and display the same features as those preformed on the donor substrate without any other sophisticated fabrication methods. Meanwhile, the materials that remain embedded in the donor substrate can also serve as functional nanostructures. The works of adhesion at the interfaces of the final tape-deposited material-donor substrate assembly are leveraged to facilitate the transfer of the deposited materials.

### Plasmonic metal nanostructures on tape

**Fig. 2:**
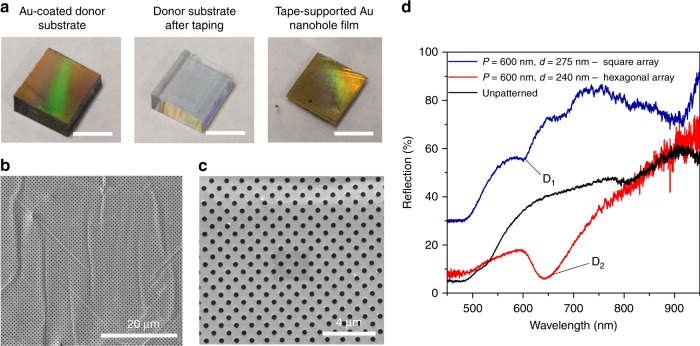
Plasmonic nanohole array on the surface of Scotch tape. **a** Photographs showing the PDMS donor substrate coated with an Au film (left), the same substrate after the Au film is stripped (middle), and the nanohole film transferred to the surface of the tape (left). Scale bars represent 2 cm. **b, c** SEM images of the stripped single-layer Au nanohole array on the tape surface. **d** Measured reflection spectra for the transferred single-layer gold nanohole array (period: *P* = 600 nm) arranged in square (hole diameter: *d* = 275 nm) and hexagonal (*d* = 240 nm) patterns. D_1_ and D_2_ in **d** show the reflection dips for the square and hexagonal spaced arrays, respectively, associated with the excitation of (1,0) order SPP at the air–Au and tape–Au interfaces

Fig. 1 illustrates the procedures for forming an array of Au nanoholes on the surface of a prepatterned PDMS donor substrate and then transferring that array to the adhesive surface of Scotch tape (3M; St Paul, MN, USA). Specifically, the PDMS donor substrate contains a periodically arranged array of nanowells fabricated from a silicon master mold using a soft lithography-based replica molding method (see the Methods section). The deposition of a 40-nm-thick Au layer onto the PDMS substrate immediately results in an array of Au nanoholes on the PDMS surface and an array of Au nanodisks at the bottoms of the PDMS nanowells. Subsequently, the Scotch tape is applied to the surface of the Au-coated PDMS substrate and then is smoothed down with one’s fingertips. Due to the poor adhesion between Au and PDMS, the Au nanohole film can be easily peeled off of the PDMS surface (Fig. 2a). Scanning electron microscopy (SEM) images (Fig. 2b, c) indicate that the Au nanohole array transfers to the tape surface with high uniformity.

Fig. 2d shows the experimental reflection spectra from the square and hexagonal spaced Au nanohole arrays transferred to the tape surface, both with periods of *P* = 600 nm. The optical resonances for the square (D_1_) and hexagonal (D_2_) lattices are observed as dips in their reflectances at wavelengths of 600 and 637 nm, respectively. These two dips are present due to the excitations of (1,0) order surface plasmon polaritons (SPP) at the air–Au and tape–Au interfaces of the square and hexagonal spaced arrays. The microscale kinks observed in Fig. 2b have a negligible influence on the overall optical properties of the transferred patterns in terms of resonance wavelength and quality factor (*Q* factor) because the SPP modes excited in the transferred nanoholes mainly depend on the angle between the incident light and the sample surface. Furthermore, atomic force microscopy measurements (see Figure S1 in the Supporting Information) show that plain Scotch tape has a surface roughness within 5 nm, and thus the surface has almost no influence on the optical properties of the nanopatterns obtained. After the Au nanohole array is transferred to the tape surface, the remaining Au inside the PDMS nanowells forms an array of 40-nm-thick Au nanodisks without using any additional patterning techniques such as lithography or etching.

**Fig. 3:**
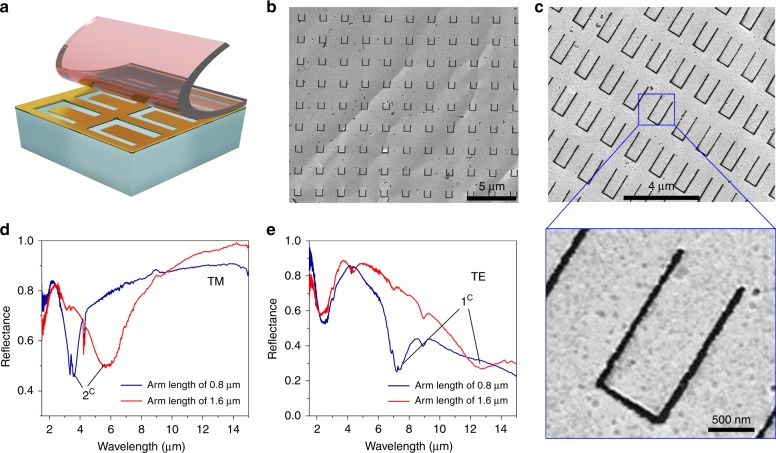
Plasmonic metamaterial of c-SRRs formed on the tape surface. **a** Schematic of a method using Scotch tape to transfer a thin Au layer deposited onto a PDMS donor substrate with preformed 340-nm-deep air-void nanoslits on its surface. **b**, **c** SEM images of the tape-supported Au complementary metamaterial. The widths of the nanoslits are ~90 nm. **d**, **e** Reflection spectra recorded for two Au complementary metamaterials. 1^C^ and 2^C^ are the first- and second-order c-SRR modes, respectively. The TM and TE mode spectra are shown in **d** and **e**, respectively

Fig. 3 shows the formation of a complementary split ring resonator (c-SRR)-based plasmonic metamaterial on the Scotch tape surface. The PDMS donor substrate used here contains an array of 340-nm-deep U-shaped nanoslits and is coated by a 40-nm-thick Au layer (Fig. 3a). The tape is applied to and peeled from the Au-coated PDMS substrate to transfer the Au nanoslit film to the tape surface. The nanoslits embedded in the Au layer serve as c-SRRs. Fig. 3b, c shows two tape-supported c-SRR arrays with arm lengths of *L* = 0.8 and 1.6 µm, respectively. The width of each of the c-SRRs is approximately 90 nm. Fig. 3d, e shows the measured reflection spectra for the two c-SRR arrays, which have conspicuous resonance dips. The even or odd c-SRR eigenmodes of c-SRR arrays are excited by transverse-magnetic (TM)- or transverse-electric (TE)-polarized fields^28^. For example, under TM polarization, the second-order c-SRR excitation mode (2^C^) of the transferred c-SRRs with *L* = 1.6 µm is observed at a wavelength of 6 µm, while the c-SRRs with *L* = 0.8 µm exhibit the 2^C^ mode at a wavelength of 3.8 µm. Under TE polarization, the first-order c-SRR mode (1^C^) for the c-SRRs with *L* = 0.8 µm appears at 7 µm, while for the c-SRRs with *L* = 1.6 µm, the 1^C^ mode redshifts to 12.6 µm. The presented technique could be applied to obtain many other air-void nanopatterns with different shapes.

Essentially, the tape transfer of the nanopatterned film from the surface of the donor substrate relies on the work of adhesion *W*_A–B_ at the interface between two contacting materials A and B, which can be described by Eq. (1):^29^

1 $$W_{{\mathrm{A}} - {\mathrm{B}}} = \pi r^2\left( {\frac{{\gamma _{\mathrm{A}}^{\mathrm d}\gamma _{\mathrm{B}}^{\mathrm d}}}{{\gamma _{\mathrm{A}}^{\mathrm d} + \gamma _{\mathrm{B}}^{\mathrm d}}} + \frac{{\gamma _{\mathrm{A}}^{\mathrm p}\gamma _{\mathrm{B}}^{\mathrm p}}}{{\gamma _{\mathrm{A}}^{\mathrm p} + \gamma _{\mathrm{B}}^{\mathrm p}}}} \right),$$

where *γ*^d^ and *γ*^p^ correspond to the dispersion and polar components of surface energy, respectively (*γ*=*γ*^d^+*γ*^p^). For example, peeling the nanopatterned Au film from the PDMS surface using the tape is possible because *W*_Au-Tape_ (68.5 mJ m^−2^) > *W*_Au-PDMS_ (52.2 mJ m^−2^). Table S1 (Supplementary Information) provides the surface energies of the materials used in this work and the *W*_A–B_ values calculated for different contacting interfaces.

### All-dielectric nanophotonic structures on tape

**Fig. 4:**
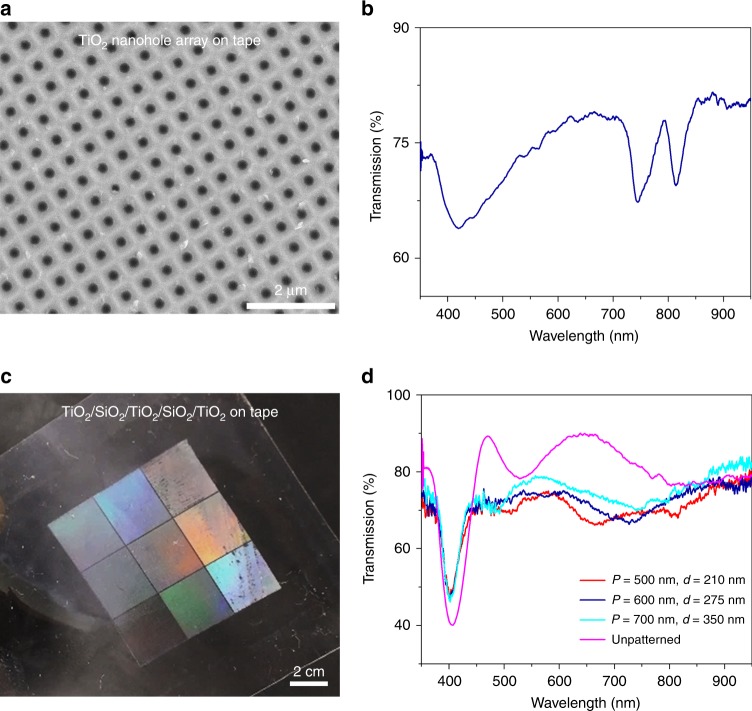
All-dielectric single- and multilayer nanohole array on a tape surface. **a** SEM image of the single-layer TiO_2_ nanohole array formed on the tape surface. The period of the array is 500 nm, and the diameter of each hole is 210 nm. **b** Transmission spectrum for the transferred TiO_2_ nanohole array shown in **a**. **c** Photographs of the transferred five-layer stack of TiO_2_/SiO_2_/TiO_2_/SiO_2_/TiO_2_ nanohole films with different periods in a total area of 8 × 8 cm^2^. **d** Transmission spectra for the transferred five-layer dielectric film stacks in **c** with different periods and hole diameters and an unpatterned reference film stack deposited on the tape surface

In addition to transferring metallic nanostructures, the tape nanolithography technique can also produce nanopatterned dielectric films on the surface of adhesive tape. Fig. 4a shows a single-layer TiO_2_ nanohole film (period: *P* = 500 nm, hole diameter: *d* = 210 nm) transferred to a Scotch tape surface showing high structural completeness and uniformity. The PDMS donor substrate used here contains 200-nm-deep nanowells and is coated by a 200-nm-thick TiO_2_ layer using e-beam evaporation. Transferring the TiO_2_ nanohole film to the tape surface is possible because *W*_TiO2-Tape_ (19.3 mJ m^−2^) > *W*_TiO2-PDMS_ (13.6 mJ m^−2^). Fig. 4b shows that the transmission spectrum of the transferred nanohole TiO_2_ film contains guided mode resonance features. The transmission dips observed for the unpatterned films originate from the multilayer thin-film interference effect. Introducing nanoholes in these films generally allows more light to transmit, as indicated by the spectral dip at a wavelength of 400 nm. However, due to the resonance of the nanoholes, the transmission intensity at the resonance wavelength decreases, as demonstrated by the spectral dip at a wavelength of 650 nm for *P* = 500 nm and *d* = 210 nm (Fig. 4d).

Further, an all-dielectric five-layer stack of TiO_2_/SiO_2_/TiO_2_/SiO_2_/TiO_2_ nanohole films is formed through sequential film depositions on the surface of a PDMS donor substrate containing nanowells. Each TiO_2_ or SiO_2_ layer is 50 nm thick. The dielectric nanohole stack obtained is then peeled off and transferred to the surface of Scotch tape (Fig. 4c). The alternating dielectric nanohole films stay together during peeling from the PDMS without treatment of the interfaces between the TiO_2_ and SiO_2_ layers because *W*_TiO2-Tape_ (19.3 mJ m^−2^) > *W*_TiO2–SiO2_ (16.6 mJ m^−2^) > *W*_TiO2-PDMS_ (13.6 mJ m^−2^). Fig. 4d demonstrates that the high refractive index contrast between the alternating layers (index: *n*_TiO2_ = 2.25, *n*_SiO2_ = 1.46) results in clear thin-film destructive interference at a wavelength of 400 nm, which also confirms that the multilayered nanohole stack transferred to the tape surface successfully.

### Metallo-dielectric nanophotonic structures on tape

**Fig. 5:**
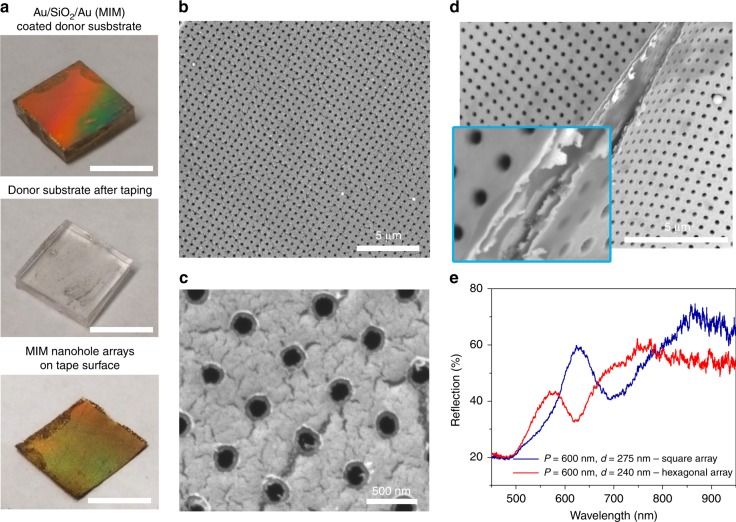
Metallo-dielectric nanophotonic structures on the surface of Scotch tape. **a** Photographs of the three-layer Au/SiO_2_/Au nanohole films before (left top) and after (left middle) taping. The bottom left photo shows the MIM nanohole arrays transferred to the tape surface. The scale bars represent 20 mm. **b**, **c** SEM images of the stripped Au/SiO_2_/Au films on the tape surface. **d** Cross-sectional view of the Au/SiO_2_/Au film. The inset shows a magnified cross-sectional image of the top and bottom Au layers. **e** Measured reflection spectra of the Au/SiO_2_/Au nanohole film on the tape surface with *P* = 600 nm arranged in square and hexagonal patterns

In addition to producing metallic or dielectric nanostructured films, the tape nanolithography method is also able to produce a tape-supported stack of metallic and dielectric nanohole films. For example, a three-layer Au/SiO_2_/Au or five-layer Au/SiO_2_/Au/SiO_2_/Au stack is deposited onto the surface of a PDMS donor substrate containing nanowells by alternating e-beam evaporation of Au and SiO_2_ layers, each with a thickness of 40 nm. Fig. 5a shows that the three-layer MIM nanohole stack can be successfully peeled from the PDMS substrate without damage because *W*_Au-SiO2_ (103.8 mJ m^−2^) > *W*_Au-Tape_ (68.5 mJ m^−2^) > *W*_Au-PDMS_ (52.2 mJ m^−2^). No remaining residues are found on the PDMS surface. Fig. 5b–d demonstrates the high structural completeness of the transferred MIM nanohole stack. The major factors that determine whether the transfer of deposited materials from the PDMS donor substrate to the tape surface is successful include (i) the interfacial bonding strength between the PDMS and the first layer of deposited material and (ii) the interlayer bonding strengths between the subsequently deposited materials. Because the bonding strength between Au and SiO_2_ is lower than that between SiO_2_ and TiO_2_, we could only obtain a three-layer stack of Au/SiO_2_/Au nanohole films, while we could successfully obtain a five-layer stack of TiO_2_/SiO_2_/TiO_2_/SiO_2_/TiO_2_ nanohole films. Fig. 5e shows the measured reflection spectra of two on-tape MIM nanohole stacks (period: *P* = 600 nm) arranged in square (hole diameter: *d* = 275 nm) and hexagonal (*d* = 240 nm) patterns. However, for the case of the five-layer MIMIM stack, many residues remain on the PDMS surface after peeling, perhaps due to high internal stress accumulated within the stack during the deposition of five layers of films on the tape surface. Nevertheless, the ability to form on-tape MIM nanohole arrays may enable the cost-efficient fabrication of flexible negative-index materials and metamaterial absorbers^15,30,31^.

### Nanophotonic structures that remain embedded inside the donor substrate

**Fig. 6:**
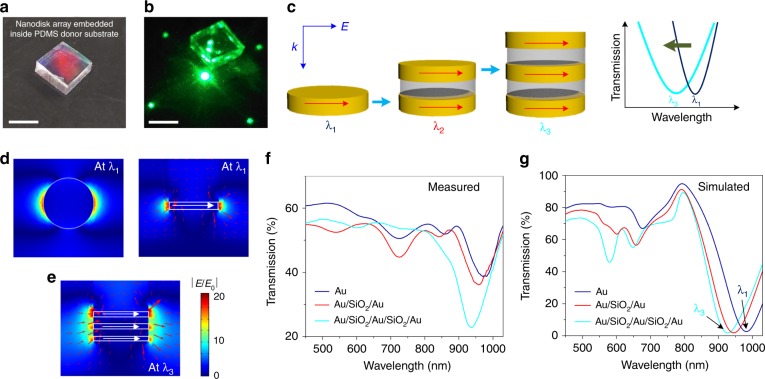
Nanodisks left behind at the bottoms of nanowells on the donor substrate with shapes complementary to the transferred nanoholes on the tape surface. **a** Photograph of the PDMS donor substrate with an embedded Au nanodisk array (period: *P* = 600 nm, diameter: *d* = 275 nm, square lattice) after the taping process. **b** Transmitted diffraction patterns of the device in **a**. The scale bars in **a** and **b** are 20 mm. **c** Schematic showing that as the number of Au nanodisk layers increases from 1 to 3, the resonance wavelength blueshifts from *λ*_1_ to *λ*_3_, while the bandwidths of the dips in the resonance increase. **d**, **e** Simulated electric field distributions for the resonance wavelengths *λ*_1_ and *λ*_3_ denoted in **g**. **f**, **g** Measured and simulated transmissions of the nanodisk arrays (*P* = 600 nm, *d* = 275 nm) with different numbers and compositions of layers

Importantly, tape nanolithography enables the easy manufacturing of single- or multiple-layered nanostructures embedded inside the donor substrate. After the tape transfer of the deposited materials, the materials left behind inside the nanowells of the donor substrate (Fig. 6a, b) have precise complementary shapes to those transferred to the tape surface. Their optical properties are also dependent on their material compositions. Fig. 6c shows a conceptual schematic for tuning the localized surface plasmon (LSP) modes of different metallo-dielectric configurations of the nanodisks that remain inside the nanowells. A single-layer Au nanodisk exhibits an LSP resonance at a wavelength of *λ*_1_. As more nanodisks are vertically stacked, for example, in the form of Au/SiO_2_/Au or Au/SiO_2_/Au/SiO_2_/Au, the resonance coupling between the multiple Au nanodisks leads to a decrease in resonance wavelength and an increase in bandwidth. Essentially, the optical resonance of the Au nanodisk array originates from the collective oscillations of the electric dipoles of each Au disk, as indicated by the electric field distributions depicted in Fig. 6d, e. Fig. 6f, g shows experimental and simulated transmission spectra, respectively, for nanodisk arrays (*P* = 600 nm and *d* = 275 nm) with different numbers and compositions of materials remaining inside the nanowells. As the number of Au nanodisk layers increases to three, the measured LSP resonance exhibits a blueshift and a bandwidth increase due to the stacking of electric dipoles, as depicted in Fig. 6e. Specifically, the resonance wavelengths for the single-layer Au nanodisks and the Au/SiO_2_/Au nanodisks blueshift from 980 to 940 nm, and the values of the full-width at half-maximum increase from 113 to 154 nm.

### Demonstration of refractive index sensing

**Fig. 7:**
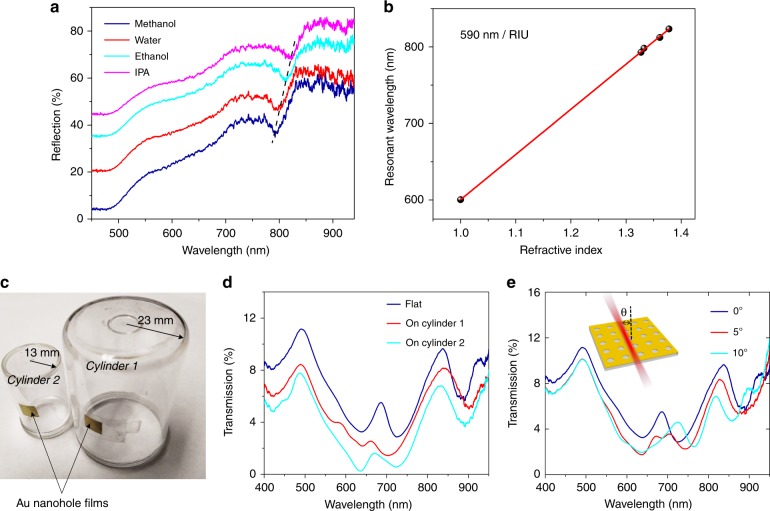
Refractive index sensing and conformal plasmonic surface. **a** Reflection spectra of the tape-supported square-lattice Au nanohole array exposed to different index media. **b** Linear fit of the resonance wavelength as a function of the ambient refractive index. **c** Photograph of the Au nanohole arrays conformably attached to curved cylinders with radii of 13 and 23 mm. **d** Transmission spectra for the two curved films shown in **c** and one flat-surface Au nanohole array, all measured under a normally incident light beam with a 4 mm diameter. **e** Transmission spectra for the flat nanohole arrays under obliquely incident light. The Au nanohole arrays examined in panels **d** and **e** are arranged in a hexagonal pattern with a period of 600 nm

The refractive index sensitivity of the tape-based, square-lattice Au nanohole array (*P* = 600 nm, *d* = 275 nm) is characterized for potential biosensor applications. The ambient refractive index of the device is varied using pure methanol (1.327), water (1.3325), ethanol (1.3612), and isopropyl alcohol (1.3777) (Sigma-Aldrich, MO, US). The tape-based Au nanohole array initially exhibits a (1,0) SPP resonance dip at a wavelength of 601 nm in air (Fig. 7a). As the ambient refractive index increases, the resonance wavelength shifts to longer wavelengths. Fig. 7b shows the linear fit of the resonance wavelength as a function of the refractive index. Based on the slope of this linear fit, the sensitivity of this mode to changes in the refractive index is 590 nm/RIU. Though the *Q* factor of the resonance may be slightly affected by nanoscale cracks introduced during the tape transfer process, the transferred nanostructures exhibit high refractive index sensitivity. With its high refractive index sensitivity, the tape-supported Au nanohole array has the potential to serve as the surface of an index-based biochemical sensor where specific analyte-ligand binding events at the metal surface lead to changes in the local refractive index in the vicinity of the nanoholes.

### Demonstration of conformal plasmonic surface

The effect of the mechanical flexibility of the tape-supported Au nanohole film on the incident light angle-dependent SPP modes is studied. As shown in Fig. 7c, Au nanohole films with a period of 600 nm arranged in a hexagonal pattern are attached to the curved surfaces of two acrylic cylinders with radii of *r* = 13 and 23 mm. When the nanohole film is curved, the transmittance of the incident light (along the radial direction) decreases, and an additional transmission peak appears at a wavelength of approximately 600 nm for the smaller cylinder. However, with further increases in the curvature, the obtained spectra assume approximately their original shapes with decreased resonance strengths. The light beam has a 4 mm diameter and is normally incident on the smaller cylinder surface (*r* = 13 mm), which is approximately equivalent to the light incident on a flat surface at an angle continuously ranging from −8.8° to 8.8° (−5.0° to 5.0° for the cylinder with *r* = 23 mm). For comparison purposes, transmission spectra are also recorded for a flat nanohole array under oblique incident angles of 5° and 10°. The changes observed in the spectra of the flat nanohole array under an increasing oblique incident angle (Fig. 7e) are in good agreement with those for the nanohole arrays with increasing curvatures (Fig. 7d). The results obtained confirm that the optical properties of the nanohole arrays supported on the curved surfaces depend on the surface curvature and that the related spectral variations can be qualitatively interpreted using a flat surface under obliquely incident light as a reference. As reported^32,33^, Au nanohole arrays sustain good optical performances under tensile strains of 7–10%. However, the Scotch tape used in our experiment is not an elastomeric substrate with a limited stretchability. Thus, to realize on-tape stretchable Au nanohole arrays, one could consider using elastomeric adhesive tapes for transferring Au nanohole arrays.

### Demonstration of a flexible F–P cavity resonator

**Fig. 8:**
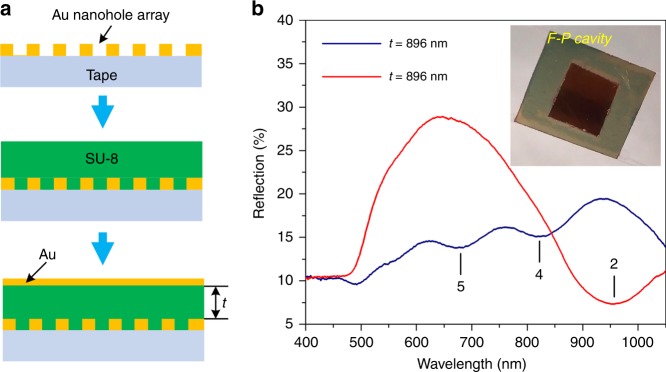
Flexible F–P cavity device formed on the tape surface. **a** Fabrication of F–P cavities. After transferring the tape lithographic Au nanoholes to the surface of Scotch tape, they are spin-coated with an SU-8 photoresist layer with thickness *t*, followed by the deposition of a 15-nm-thick Au layer. **b** Reflection spectra for the F–P cavities formed on the tape surface with different cavity lengths under normally incident light. The studied Au nanohole array is arranged in a hexagonal pattern with a period of 600 nm and a hole diameter of 240 nm. The numbers labeling the resonance dips correspond to the order of the F–P modes. The inset shows a photograph of the fabricated F–P cavity

The tape lithographic nanostructures can be used as key components for some flexible optical devices. Fig. 8a shows an F–P cavity resonator realized on the tape surface. To produce this optical resonator, an Au nanohole film is first formed on double-sided Scotch tape using the tape nanolithography technique. The tape is then attached to the surface of a 500-µm-thick PDMS layer spin-coated onto a silicon wafer. Subsequently, an SU-8 photoresist layer (which determines the cavity length *t*) is spin-coated onto the surface of the tape-supported Au nanohole film, followed by the deposition of a 15-nm-thick Au layer. Therefore, an F–P cavity resonator is realized on the double-sided tape and then peeled from the PDMS surface (see the inset in Fig. 8b). Fig. 8b shows the reflection spectra for two F–P devices with cavity lengths of 896 and 453 nm. At a cavity length of 896 nm, the four-order F–P mode measured agrees with the predicted wavelength of 800 nm (estimated using the reflection coefficients derived in refs. ^34,35^), and the higher orders of the F–P resonance (fifth-order mode at 675 nm) are also observed in the measured wavelength range. For the device with a cavity length of 453 nm, the second-order F–P mode is calculated at a wavelength of 950 nm, which almost agrees with the observed dip at the measured 960 nm wavelength shown in Fig. 8b. Notably, the performance of the flexible F–P cavity is sensitive to the surface quality of the transferred Au nanohole film. During transfer, some cracks are introduced into the Au film (see Figure S1 in the Supporting Information). These cracks could cause optical losses inside the cavity, thus reducing the *Q* factor of the cavity. The formation of nanocracks can be minimized by lowering the bonding strength between the surface of the PDMS donor substrate and the Au through appropriate treatment of the PDMS surface before Au deposition.

## Discussion

As demonstrated above, the tape nanolithography technique provides a sub-hundred-nanometer resolution of nanopatterns in single- and multilayer metallic and dielectric thin films to realize various nanophotonic structures and devices on adhesive tapes. The high flexibility of this method in controlling the number of thin-film layers and their material compositions allows tuning of the optical properties of plasmonic, dielectric, and metallo-dielectric optical devices. Additionally, tape nanolithography can produce high-resolution nanopatterns on a donor substrate with shapes complementary to the transferred nanopatterns on the tape surface with the same material compositions.

Compared with other nanopatterning methods, our tape nanolithography method is advantageous due to a lower dependence on expensive equipment, a shorter turnaround time, and a relatively larger processing area. Specifically, this method is easy to implement with the help of appropriate donor substrates with preformed air-void features such as nanowells and nanoslits. Thanks to soft lithography, producing polymer donor substrates with customized features is relatively easy. In addition, various film deposition technologies (e.g., chemical vapor deposition and sputtering) can be directly applied to deposit different materials (e.g., metals, dielectrics, and 2D materials) on the surface of a donor substrate to form nanopatterns in the material without using any complicated patterning techniques. When the thin-film materials require high deposition temperatures unfavorable for the donor substrate, this issue can be mitigated by choosing a material that is durable at high temperatures for the donor substrate. Moreover, the donor substrate can be reused until the air-void patterns on the donor substrate are filled with deposited materials. Notably, the transfer of a nanopatterned film with an area of multiple square inches can be completed within 1 min after the thin-film materials are deposited.

**Fig. 9:**
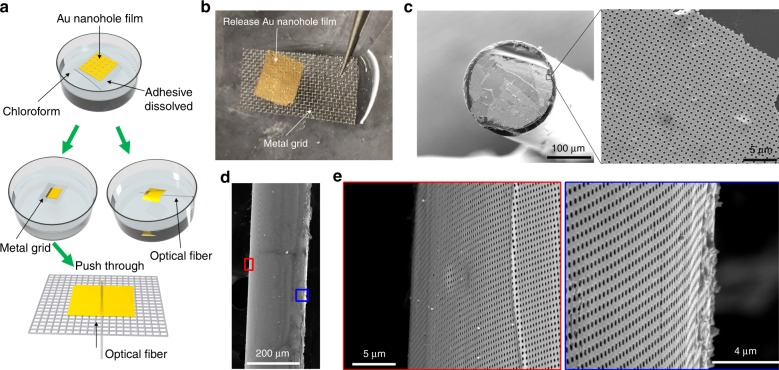
Tape nanolithography-assisted formation of a standalone Au nanohole film and its transfer to an optical fiber surface. **a** Schematic of the release-and-transfer process of the Au nanohole film to the optical fiber surface. **b** Photographs of the released Au nanohole film, which is then supported by a metal grid substrate. **c** SEM images of the Au nanohole film transferred to the fiber facet. **d**, **e** SEM images of the standalone Au nanohole film wrapped on the sidewall of the fiber. The locations of the two images taken on the fiber in **e** are shown in the red and blue windows marked in **d**

Interestingly, tape nanolithography also enables the formation of a standalone nanopatterned film by dissolving the tape and the transfer of the released film to a target surface of interest. Fig. 9a illustrates the process for transferring a tape lithographic Au nanohole film to a cleaved facet or curved side of an optical fiber. First, the tape-supported Au nanohole film is immersed in chloroform (Sigma-Aldrich, St. Louis, MO) for approximately 2 min to release the Au film. Subsequently, pure ethanol (Sigma-Aldrich, St. Louis, MO) is used to replace the chloroform and rinse the Au film. Next, the suspended Au film is picked up by a metal grid with a square hole size of 0.5 × 0.5 mm^2^ (Fig. 9b). To transfer the Au film to a multimode optical fiber (210 µm diameter; Thorlabs, Newton, NJ), the tip of the fiber is directly pushed through a hole in the metal grid across which the Au film is supported. As a result, the part of the Au film that is in contact with the fiber tip is transferred to the cleaved fiber facet (Fig. 9c). The Au nanohole array is incomplete at the edge of the fiber facet due to being pushed through the metal grid hole. Alternatively, the suspended Au film is lifted from the ethanol solution using the optical fiber, and thus the Au film is attached to the curved side of the fiber (Fig. 9d, e). Many existing patterning techniques for producing nanopatterns on a fiber facet rely on various lithographical^36,37^ or self-assembly methods^38,39^, which are relatively expensive or difficult to perform. Nanoskiving^40^ is also able to transfer nanopatterns to a fiber facet, but a critical part of this method is the sectioning of nanostructures embedded in thin epoxy slabs, which is time-consuming. In contrast, the tape nanolithography-assisted formation and transferring of standalone and wrapped nanopatterned films is relatively simple and fast.

## Conclusions

In summary, we have explored a simple tape nanolithography method for realizing a variety of flexible nanophotonic structures on the surface of a tape and complementary-shaped nanopatterns embedded inside the donor substrate. These nanostructures have sub-hundred-nanometer resolutions. The nanopatterns preformed on the PDMS donor substrate are directly transferred to a single-layer film or a stack of multiple-layer films after the material deposition, thus eliminating the need for any complicated nanopatterning techniques. A simple stick-and-peel process is used to transfer the nanopatterned materials from the donor substrate to the tape surface. We have developed tape-based single-layer metallic plasmonic nanohole arrays and complementary metamaterials with nanoslits, all-dielectric five-layer TiO_2_/SiO_2_/TiO_2_/SiO_2_/TiO_2_ nanohole films, and metallo-dielectric Au/SiO_2_/Au-based MIM nanohole structures, all of which validate the strong ability of the tape nanolithography method to tune the geometrical and material configurations and thus the optical properties of the nanopatterns. Further, we have studied the refractive index sensitivity of a tape-based plasmonic nanohole array, the angle dependence of the SPP mode of the conformable plasmonic surface, and the cavity behavior of the flexible F–P resonator, which demonstrate the possibilities for using tape lithographic nanostructures in a variety of applications. Due to the ability to easily form nanopatterns in various films and transfer the nanopatterned films to planar or irregularly shaped surfaces of different target substrates, many applications will be found for the present nanomanufacturing technique, such as flexible transformation optics;^41^ physical, chemical and biological sensing;^42–44^ and wearable nanophotonics^45,46^.

## Materials and methods

### Fabrication of PDMS donor substrates

A soft lithography-based replica nanomolding process is used to produce all the PDMS donor substrates. In this step, silicon-based master molds are manufactured using e-beam lithography and deep reactive ion-etching methods. Each silicon mold is silanized with a drop of (tridecafluoro-1,1,2,2-tetrahydrooctyl)-1-trichlorosilane (T2492-KG; United Chemical Technologies, Bristol, PA, USA) in a vacuum desiccator under active vacuum for 20 min. Subsequently, a PDMS solution and its corresponding curing agent (Sylgard 184; Dow Corning, Auburn, MI, USA) are mixed at a weight ratio of 10:1 and degassed in the vacuum desiccator for 20 min. The PDMS mixture is then poured onto the top surface of the silicon mold and cured on a hotplate at 65 °C for 2 h. Then, the PDMS stamp is peeled from the silicon mold.

### Electron beam evaporation

A Temescal electron beam evaporator (BJD-1800) is used to deposit Au, SiO_2_, and TiO_2_ thin films onto the surfaces of PDMS donor substrates. The chamber pressure of the system is approximately 1 × 10^−6^ Torr.

### Optical measurements

Transmission and reflection spectra for the samples on the tape surfaces are measured and recorded using a spectroscopic measurement setup. A white light emitted from a 150 W quartz halogen lamp is coupled with a multimode fiber collimated by an objective lens and directed to the sample surface. The reflection spectra of the c-SRR array samples on the tape surfaces are recorded via Fourier transform infrared spectroscopy (Hyperion 2000; Bruker, Billerica, MA, USA) under normally incident light.

### Optical simulations

The optical simulations are performed using finite element method-based software (COMSOL Multiphysics; COMSOL, Stockholm, Sweden). The geometrical parameters used in the simulations are obtained from the SEM images of the studied samples.

## Electronic supplementary material

Supporting Information
