# Supplementary material for: Tape nanolithography: a rapid and simple method for fabricating flexible, wearable nanophotonic devices
Source: Microsyst Nanoeng. 2018 Oct 8;4:31. doi: 10.1038/s41378-018-0031-4 (PMC6220255; doi:10.1038/s41378-018-0031-4)
Supplement: Supplementary file 1 — Supporting Information [file 41378_2018_31_MOESM1_ESM.pdf]

Supplementary Information

Tape Nanolithography – A Rapid and Facile Method to Fabricate Flexible and Wearable Nanophotonic Devices

Qiugu Wang[1], Weikun Han[1], Yifei Wang[1], Meng Lu[1,2] and Liang Dong[1,3*]

[1]Department of Electrical and Computer Engineering, Iowa State University, Ames, Iowa 50011, USA

[2]Department of Mechanical Engineering, Iowa State University, Ames, Iowa 50011, USA

[3]Microelectronics Research Center, Iowa State University, Ames, Iowa 50011, USA

[*]Corresponding author: L.D. Email: ldong@iastate.edu; Phone: +1-515-294-0388

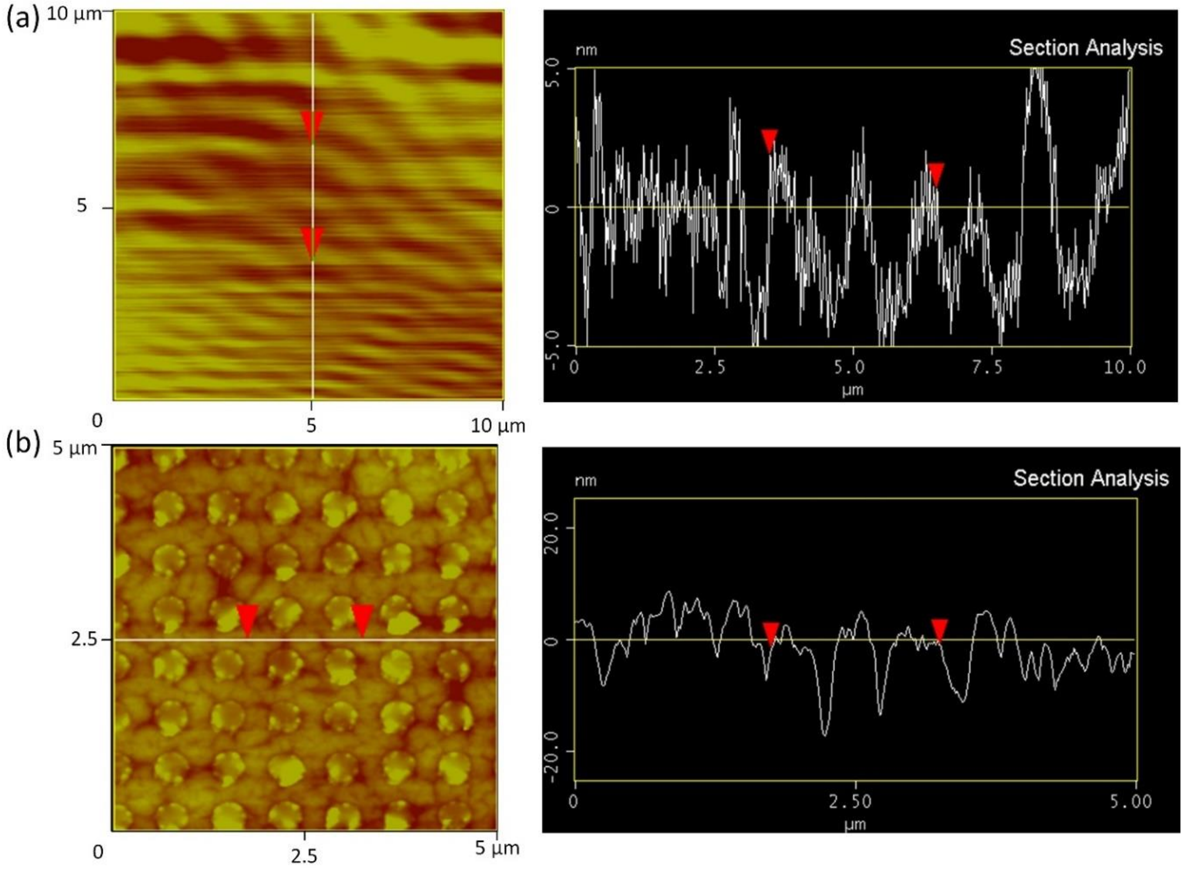

Figure S1 (Left column) AFM images of Scotch tape (a) and transferred Au nanohole arrays on the tape (b). (Right column) The corresponding section analysis of intersectional lines as indicated in AFM images.

Figure S1 shows atomic force microscopy (AFM) measurements of the plain Scotch tape and the transferred Au films with the Au nanohole arrays transferred onto the tape. The plain tape in Fig. S1 exhibits some wrinkles with surface roughness within 5 nm. After the tape transfer of nanohole arrays, some nanocracks are observed. A plausible way to minimize the formation of nanocracks may be to decrease the bonding strength between PDMS and Au through appropriate surface treatment of PDMS before Au deposition.

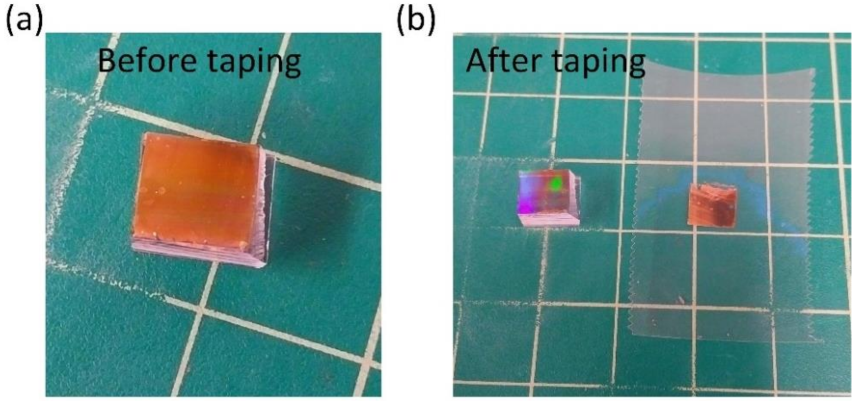

Figure: (a) Photograph of an PDMS mold with an array of nanoposts covered by a 40-nm-thick Au layer. (b) Photograph of the mold in (a) and Scotch tape after taping.

In Figure S2, we utilized the taping method to transfer Au nanodisks (40-nm-thick and 200-nm-diameter) from the top surface of PDMS-based circular nanopillar arrays (250-nm-deep and 200-nm-diameter) onto Scotch tape surface. However, because the applied pressure deforms the PDMS nanopillars during the transferring process, the tape was pressed in contact with the continuous Au film on the PDMS surface. As a result, part of the Au film is also transferred onto the tape surface, in addition to the transfer of the Au nanodisks.

Table S1: Surface energies of different materials[1] and work of adhesion at the interfaces between two contacting materials utilized in this work

Materials	Surface energy ($mJ\cdot m^{-2}$)			Work of adhesion ($mJ\cdot m^{-2}$)
	γ	γ^{d}	γ^{p}	$W_{A\text{-}B}$
PDMS	19.8	19	0.8	
Au	46.8	34.9	11.9	
Scotch® tape (Rubber resin)	33.6	33.6	0	
Si	65.1	29.9	35.2	
SiO_2	71.9	21.9	50	
TiO_2	43.9	41.5	2.4	
Au–PDMS				52.2
Au– Scotch® tape (Rubber resin)				68.5
Au–Si				49.6
Au-SiO_2				103.8
TiO_2-SiO_2				16.6
SiO_2– Scotch® tape (Rubber resin)				53.0
SiO_2– PDMS				43.8
TiO_2– Scotch® tape (Rubber resin)				19.3
TiO_2– PDMS				13.6

1. Kim, H., Yoon, B., Sung, J., Choi, D. G. & Park, C. Micropatterning of thin P3HT films via plasma enhanced polymer transfer printing. *J. Mater. Chem.* **18**, 3489-3495 (2008).
